# Plain packaging of cigarettes and smoking behavior: study protocol for a randomized controlled study

**DOI:** 10.1186/1745-6215-15-252

**Published:** 2014-06-25

**Authors:** Olivia M Maynard, Ute Leonards, Angela S Attwood, Linda Bauld, Lee Hogarth, Marcus R Munafò

**Affiliations:** 1 MRC Integrative Epidemiology Unit, University of Bristol, Oakfield House, Oakfield Grove, Bristol BS8 2BN, UK; 2 UK Centre for Tobacco and Alcohol Studies, Clinical Sciences Building, City Hospital, Nottingham NG5 1PB, UK; 3 School of Experimental Psychology, University of Bristol, 12a Priory Road, Bristol BS8 1TU, UK; 4 Institute of Social Marketing, University of Stirling, Stirling FK9 4LA, UK; 5 Department of Psychology, College of Life and Environmental Sciences, University of Exeter, Washington Singer Building, Perry Road, Exeter EX4 4QG, UK

**Keywords:** Smoking, Plain packaging, Standardized packaging, Randomized controlled trial, Health warnings, Smoking behavior

## Abstract

**Background:**

Previous research on the effects of plain packaging has largely relied on self-report measures. Here we describe the protocol of a randomized controlled trial investigating the effect of the plain packaging of cigarettes on smoking behavior in a real-world setting.

**Methods/Design:**

In a parallel group randomization design, 128 daily cigarette smokers (50% male, 50% female) will attend an initial screening session and be assigned plain or branded packs of cigarettes to smoke for a full day. Plain packs will be those currently used in Australia where plain packaging has been introduced, while branded packs will be those currently used in the United Kingdom. Our primary study outcomes will be smoking behavior (self-reported number of cigarettes smoked and volume of smoke inhaled per cigarette as measured using a smoking topography device). Secondary outcomes measured pre- and post-intervention will be smoking urges, motivation to quit smoking, and perceived taste of the cigarettes. Secondary outcomes measured post-intervention only will be experience of smoking from the cigarette pack, overall experience of smoking, attributes of the cigarette pack, perceptions of the on-packet health warnings, behavior changes, views on plain packaging, and the rewarding value of smoking. Sex differences will be explored for all analyses.

**Discussion:**

This study is novel in its approach to assessing the impact of plain packaging on actual smoking behavior. This research will help inform policymakers about the effectiveness of plain packaging as a tobacco control measure.

**Trial registration:**

Current Controlled Trials ISRCTN52982308 (registered 27 June 2013).

## Background

In countries such as the United Kingdom (UK), where all other marketing channels are prohibited, the cigarette pack is the only way in which the tobacco industry can promote their products. Plain packaging would require all cigarettes to be sold in packs with a standard pack shape, colour, and method of opening, removing all branding and leaving only the brand name in a standard font and location. In December 2012 Australia was the first country in the world to introduce plain packaging and other countries such as the UK and New Zealand are either considering or are committed to doing the same. Research shows that plain packaging makes the cigarette pack less appealing both in terms of the pack itself [1-3] and the taste and quality of the cigarettes inside [4-6], prevents the use of misleading pack characteristics [7], and increases attention to cigarette pack health warnings [8,9]. A systematic review of the literature was published in 2011 [10].

Research has also shown that using plain packs in real-world settings increases avoidance and cessation behaviors [2,3]. The first study to investigate the effect of plain packaging in a real-world environment was a pilot study where 48 daily smokers were required to smoke cigarettes in plain packs for two weeks and in their regular branded packs for two weeks, using a counterbalanced design [2]. In a larger follow-up study, 187 female smokers used plain and branded packs of cigarettes for one week each [3]. In both studies participants completed a questionnaire twice a week assessing perceptions of the pack, attitudes to smoking, the salience of the health warnings, and smoking-related behaviors. Almost all participants reported that the plain packaging either reduced their cigarette consumption or increased their avoidant behavior towards smoking and the pack itself. These effects were most pronounced among female smokers in the first study. These studies are the first to assess the impact of using plain cigarette packaging on attitudes to smoking in a real-world setting, although they are limited by their reliance on self-report measures. The impact of plain packaging on smoking behavior in a real-world environment using an experimental design is therefore yet to be investigated.

### Study objectives and hypotheses

We propose a randomized controlled trial to assess the impact of plain packaging compared to branded packaging on cigarette smoking behavior over the course of a typical smoking day. We hypothesize that those participants randomized to receive plain packs compared to branded packs will smoke fewer cigarettes and will have reduced exposure to cigarette smoke over the course of the day. We will explore whether these effects are most pronounced among female smokers.

Similar to the previous real-world studies, we will also assess participants’ perceptions of the cigarette packs and the cigarettes they contain, and their attitudes to smoking, using self-report measures. We hypothesize that participants in the plain packaging condition will report more negative perceptions of the pack, the cigarettes and their smoking experience than those in the branded packaging condition. Again, we will explore whether these effects are most pronounced among female smokers.

Finally, we will assess the effects of cigarette packaging on the reward value ascribed to tobacco. First, participants will complete a concurrent choice task where they are required to choose between earning two distinct rewards: tobacco and chocolate [11]. Those in the branded pack condition will earn points for their branded pack cigarettes, while those in the plain pack condition will earn points for their plain pack cigarettes. Preferential selection of the tobacco key in this task has been demonstrated to reflect the relative value ascribed to the tobacco versus the chocolate reward. Second, in a transfer task, participants will choose between the two rewards after being presented with either a picture of a branded or plain pack of cigarettes, or no stimulus. Typically, cigarette cues enhance the probability of tobacco choice by approximately 15% [11,12]. We will explore whether branded versus plain packages differ in their capacity to enhance tobacco choice and whether this difference is modulated by the 24-hour exposure to either the branded or plain pack. Any change in the effectiveness of these cues to motivate tobacco choice would arguably be driven by learning about these cues over the 24-hour exposure period.

## Methods/Design

### Design

This study will examine the effects of plain versus branded cigarette packaging on smoking behavior and experiences of smoking among a sample of adult daily smokers. Participants will attend a first testing session and complete baseline measures of urges to smoke and desire to quit, as well as undertaking a blind taste test of two cigarettes taken from a UK (branded) and Australian (plain) pack of cigarettes. The purpose of this taste test is to establish baseline ratings of the two cigarette types in a setting where participants have no knowledge of pack origin. Participants will be randomized to receive a branded or plain pack of cigarettes to smoke for the smoking test day, with the randomization stratified by sex. The following day, participants will smoke cigarettes from the pack assigned to them and their smoking behavior will be measured using a hand-held smoking topography monitor (CReSS; Borgwaldt KC, Richmond Virginia, United States). Participants will return for a second testing session two days after the first session, complete questionnaires regarding their experience of smoking cigarettes from the pack given to them, and complete a task assessing the rewarding value of cigarettes.

### Participants and recruitment

We will recruit regular daily smokers (*n* = 128) from the staff and students at the University of Bristol and the general population. Participants will be recruited through existing email lists, poster and flyer advertisements, online, and by word of mouth. Prior to attending the first laboratory session, participants will complete an online screening questionnaire to assess eligibility for the study based on the inclusion and exclusion criteria.

To meet the inclusion criteria, all participants must meet be daily cigarette smokers (between five and 20 cigarettes per day and smoking within one hour of waking), predominantly smoke one of the specific brands of cigarettes available in the study (Marlboro Gold, Marlboro Red, Dunhill Red, Benson and Hedges Gold, Benson and Hedges Silver), be aged between 18 and 40-years-old, have English as their first language or have an equivalent level of fluency and be able to give informed consent as judged by the lead researcher. Individuals who are not in good physical or mental health, are currently taking psychiatric medication (such as antidepressants) or illicit drugs (except cannabis), are pregnant, or are planning on stopping smoking in the next month, will not be eligible for inclusion.

Eligible participants will be contacted via telephone to arrange testing times. Eligible participants will attend two testing sessions approximately 48 hours apart. To avoid demand characteristics, participants will be informed that the study is concerned with examining smokers’ experiences of smoking over a 24-hour time period, rather than explicitly disclosing that the main aim is to evaluate the effect of plain cigarette packaging on smoking behavior. On completion of the study sessions, participants will be fully debriefed and reimbursed £30 for their time and expenses.

### Sample size determination

The sample size for the study has been calculated based on the volume of smoke inhaled by participants, which is one of the primary outcome measures. Previous data collection using these topography monitors from our laboratory indicates a mean inhaled volume per cigarette of 500 ml (SD 100). Therefore, in order to detect a reduction in inhaled volume of 50 ml per cigarette (equivalent to one fewer cigarette per day for a 10-a-day smoker) with 80% power at an alpha level of 5%, we will recruit 128 participants. Given the short-term nature of this study, we expect the number of withdrawals to be low.

### Ethical considerations and informed consent

Ethics approval from the University of Bristol Faculty of Science Research Ethics Committee has been granted (approval code: 310113607). The study will be conducted according to the 2013 Declaration of Helsinki and Good Clinical Practice guidelines. Written informed consent will be obtained from all participants prior to testing. The researcher will explain the nature and purpose of the study to the participant. The participant will receive the information sheet and will be given sufficient time to read this, consider any implications, and raise any questions with the investigators prior to making a decision to participate. Participants will be informed that they are free to withdraw from the study at any time.

### Randomization

Participants will be randomized to receive either plain or branded cigarettes to smoke for the 24-hour test day. Randomization will be stratified by sex, with equal numbers of males and females randomized to the branded and plain conditions. To ensure the lead researcher is blind to the pack condition assigned to participants at randomization, the lead researcher will contact an experimental collaborator with the participant’s preferred brand of cigarettes and the participant’s sex. Using the random number assignment software ‘Research Randomizer’ [13] and a pre-assigned code, an experimental collaborator, with no direct contact with study participants, will determine whether the participant is to be randomized to the branded or plain pack condition. The experimental collaborator will then place the correct pack into a concealed envelope labelled with the participant’s identification number. The participant will be instructed not to open the envelope and reveal the pack of cigarettes until the morning of the 24-hour test day.

In addition, the cigarettes assigned to participants to smoke in the cigarette taste test during Test Day 1 will be randomized as such that participants smoke either a cigarette from a branded or plain pack first. Both cigarettes will be from the participants’ preferred brand (for example, a UK Marlboro Gold and an Australian Marlboro Gold). In order for the lead researcher to be blind to which cigarettes are presented, the experimental collaborator will assign branded and plain packs a code of either ‘A’ or ‘B’. The experimental collaborator will place one cigarette from the participant’s usual UK pack and one from the Australian plain pack in sealed, concealed envelopes labelled with ‘A’ and ‘B’ as appropriate. To ensure that the order in which the taste test is administered is counterbalanced, participants with an odd participant number will smoke the cigarette from the envelope labelled ‘A’ first, whereas those with even participant numbers will smoke the cigarette from the envelope labelled ‘B’ first. To ensure that blinding is maintained, the lead researcher will not be present in the room while participants complete the Test Day 1 taste test.

## Materials

### Cigarette packs

**Figure 1:**
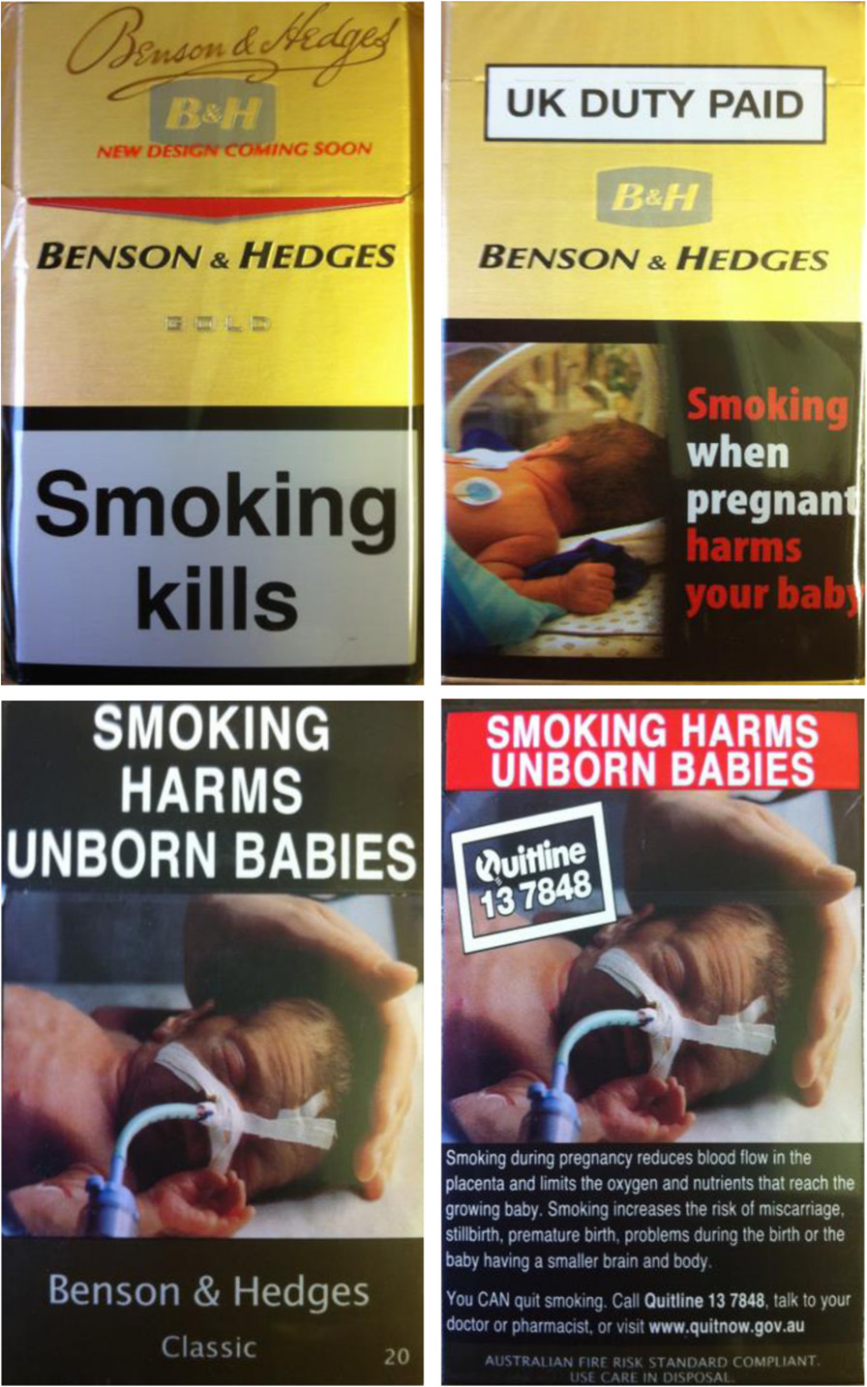
**Examples of UK branded and Australian plain cigarettes used.** The front and back of the cigarette packages are shown in both cases.

Cigarette packs provided to participants for the 24-hour pack exposure will be either branded UK packs of cigarettes, or plain Australian packs of cigarettes. These packs will comprise the ‘branded’ and ‘plain’ experimental conditions. Table 1 shows the UK branded packs used and their Australian plain pack equivalents.Since they originate from different countries packs will differ in the shape, size, and format of the health warning, however, they will be selected so that both will have an image of a baby suffering from prenatal tobacco exposure. UK pictorial warnings cover 40% of the lower half of the back of cigarette packs, whilst Australian pictorial warnings cover 75% of the front and 90% of the back of cigarette packs. Examples of the UK branded and Australian plain pack cigarettes used in this study are shown in Figure 1.

**Table 1:** UK branded cigarette packs and Australian plain pack equivalents used in the current study

**UK branded pack**	**Australian plain pack**
Marlboro Gold	Marlboro Gold
Marlboro Red	Marlboro Red
Benson & Hedges Gold	Benson & Hedges Classic
Benson & Hedges Silver	Benson & Hedges Smooth
Benson & Hedges Silver	Benson & Hedges Fine
Dunhill Red	Dunhill Premier Red

### Topography monitor

A CreSS Pocket topography monitor (CReSS; Borgwaldt KC, Richmond Virginia, United States) will be used to assess participants’ smoking behavior. This battery-operated, hand-held monitor will record for each cigarette smoked: the date, time, start and end of smoking, puffs per cigarette, puff volume, and puff duration. The ambulatory monitoring of cigarette smoking topography has been shown to generate extremely high quality data [14].

### Procedure

#### Test Day 1

On arrival at the first testing session, participants will re-read the information sheet and provide informed consent. Participants will report the number of cigarettes smoked the previous day and the number of minutes since their last cigarette. Participants smoking fewer than five cigarettes the previous day will be ineligible to participate in the study. A breath carbon monoxide test will be administered to verify recent smoking status. Participants will complete baseline questionnaires regarding contemplation of quitting smoking (Contemplation Ladder) [15], smoking urges (Questionnaire of Smoking Urges; QSU-brief) [16] and nicotine dependence (Fagerström Test for Nicotine Dependence; FTND) [17].

Participants will then complete a cigarette ‘taste test’. As per the randomization procedure, participants will be presented with two cigarettes to smoke, one from a plain pack of cigarettes, the other from a branded pack. Taste ratings at this stage of the experiment, where participants are blind to the pack the cigarettes originated from, will be compared to taste ratings completed when participants know whether the cigarettes are from a branded or plain pack at Test Day 2. In a purpose-built smoking laboratory comprising ventilated smoking cubicles, participants will be asked to take three puffs from each of the cigarettes and record their perceptions of the cigarette on a taste test questionnaire administered on paper. Participants will be required to report on a seven-point Likert scale from ‘strongly disagree’ to ‘strongly agree’ the degree to which ‘the taste of the cigarette is strong/harsh/dry/stale/dull/dirty’. Participants will also report on a seven-point Likert scale from ‘much better’ to ‘much worse’ how the cigarette compares to their normal cigarette. Participants will have five minutes to cleanse their palate with a glass of water and complete their ratings and will then complete the same procedure with the second cigarette.

As described in the randomization procedure, participants will then be provided with either a plain or a branded pack of cigarettes in a sealed envelope and a topography monitor. The envelope will include instructions of how to use the device and the phone number of the lead researcher, which should be used in case of any problems. Participants will be given verbal instructions of how to use the topography monitor and will then practice using the monitor with a cigarette in the smoking laboratory. The lead researcher will remain blind to the pack provided to the participant until the participant returns on Test Day 2, and the participant will be blind to the pack type until the following morning when they open the envelope. Participants will not be informed that the purpose of the study is to investigate the differences in smoking behavior according to pack type and participants will not be informed that the cigarette pack is an integral aspect of the experiment.

### 24-hour pack exposure

Participants will be requested to smoke only cigarettes given to them from the experimenter, through the monitor provided, for the entire day.

#### Test Day 2

Participants will return any unsmoked cigarettes (to verify the number smoked) and the smoking topography monitor, and will complete the Contemplation Ladder [15] and QSU-brief [16]. To verify the data obtained from the topography device, participants will self-report the number of cigarettes they smoked during the 24-hour pack exposure, details of any cigarettes not smoked from the pack given to them or through the monitor, and any unusual events about the previous day (other than taking part in the experiment) which may have changed their smoking behavior.

Participants will then rate the taste of only the cigarettes they were provided with (using the same questions as on the taste-test on Test Day 1) and complete questions taken from the previous study assessing real-world smoking behavior using plain packaging [2], which will include reporting their experience of using the topography monitor, experiences of smoking from the cigarette pack, experiences of smoking in general, attributes of the cigarette pack and perceptions of the on-packet health warnings (all on a five-point Likert scale), behavior changes (a series of binary questions), and views on plain packaging legislation (on a four-point Likert scale). Different scale sizes were used to replicate the scales used in previous experiments.

Participants will then complete the cigarette reward task. The initial concurrent training stage will begin with the on-screen instructions: ‘This is a game in which you can win the cigarettes and chocolate in front of you. In each trial, press the D or H key to see if you have won a point for these rewards. You will only win on some trials. Press the space bar to begin’. Crucially, whereas those participants randomized to the branded pack condition will be presented with an unopened branded cigarette versus two 49 g Cadbury Dairy Milk chocolate bars to choose between (placed on the desk in front of them), those randomized to plain packaged cigarettes will be presented with their plain cigarettes versus the same chocolate to choose between. Each trial will begin with the centrally presented text, ‘Select a key’, which will remain until either the D or H key is pressed. Pressing one key will immediately present the outcome text ‘You win one tobacco point’, whereas the other key will produce the outcome text ‘You win one chocolate point’. The key-reward assignment will be counterbalanced between participants. Each key will have a 50% chance of yielding its respective outcome. On non-rewarded trials, the outcome text ‘You win nothing’ will be presented.

Preferential selection of the tobacco key in this concurrent task has previously been demonstrated to reflect the relative value ascribed to the tobacco versus the chocolate reward. If plain packaged cigarettes are ascribed a lower value than branded cigarettes, we should find reduced tobacco choice in the plain versus branded group. There will be 40 trials of concurrent training in total. At the end of this training participants will be tested for knowledge of the instrumental contingencies through the on-screen questions: ‘Which key earned tobacco/chocolate, the D or the H key? Please choose carefully’. The order of the two questions will be randomized. Participants will then complete a transfer task in which they will choose between the tobacco and chocolate key in the presence of three cue conditions: no stimulus, plain pack, or branded pack. There will be 60 trials in total, blocked into 10 cycles of six trials, presenting two of each of the three cue conditions in random order. In trials where a pack is presented, this pack will be randomly sampled from a set of 100 (comprised of 10 brands × 10 health warnings). The difference between branded versus plain packs in their capacity to enhance tobacco choice, and whether this difference is modulated by the cigarette pack assigned to participants in the 24-hour exposure will be assessed.

Participants will finally be asked about their views on cigarette packaging and plain packaging. On completing the experiment, participants will be fully debriefed and given the opportunity to ask questions. Participants will be reimbursed with £30 for participating in the study.

### Outcome measures

Primary outcome measures will be: (1) smoking behavior across a 24-hour period, defined by the number of cigarettes smoked (measured by self-report and returned cigarettes) and (2) the average volume of smoke inhaled per cigarette (measured by the smoking topography device). Secondary outcomes measured pre- and post-intervention will be smoking urges (as measured by the QSU-brief), motivation to quit smoking (as measured by the Contemplation Ladder), and perceived taste of the cigarettes. Secondary outcomes measured post-intervention, only by self-report, will be experience of smoking from the cigarette pack, overall experience of smoking, attributes of the cigarette pack, perceptions of the on-packet health warnings, behavior changes, views on plain packaging, and the rewarding value of smoking (as measured by the concurrent choice and transfer task).

### Data analysis

We will use linear regression to evaluate the effect of cigarette packaging (branded or plain) on the primary and secondary outcome measures using an intention-to-treat analysis. We will run these analyses with and without adjustments for age, sex, heaviness of smoking, and where appropriate, corresponding baseline measures. We will further explore whether sex modifies these effects by including appropriate interaction terms in our models. As noted above, we expect the number of withdrawals from the study to be low. Missing data is most likely to occur due to failure of the smoking topography device. Based on prior experience, this is uncommon and equally likely to occur in both arms of the study.

The concurrent choice and transfer data will be analyzed in two stages. First, a 2 (condition: plain, branded) × 2 (sex: male, female) analysis of variance (ANOVA) will be conducted on the percent choice of the tobacco key in the concurrent training stage to determine if there is a reduced value ascribed to plain versus branded cigarettes. Second, a 2 (condition: plain, branded) × 2 (sex: male, female) × 3 (transfer task cue exposure condition: plain, branded, control) mixed-model ANOVA will examine whether the effectiveness of these cues in motivating tobacco-seeking behavior is influenced by 24-hour exposure to packaging. Interactions will be explored using *post-hoc* tests corrected for multiple comparisons using the Bonferroni method.

## Discussion

There are several potential strengths of our research design. First, this is the first study to examine the effect of plain packaging on actual smoking behavior (number of cigarettes smoked and smoke exposure) in a real-world setting. Previously, two studies have required smokers to use plain packs of cigarettes in a real-world setting, although these studies relied on self-report of smoking behavior and did not ask participants to report the number of cigarettes smoked [2,3]. Second, this is the first study to use genuine plain packs of cigarettes. Previous real-world studies required smokers to transfer their cigarettes into plain packs created by the researchers, a limitation recognized by the authors [3]. Although the quality of the plain packs used in these studies was good, they did not include the foil inside the cigarette pack or the plastic wrap around the pack which the smoker removes when opening the cigarette pack. These elements add to the experience of smoking and it is possible that, as compared with the tobacco industry manufactured packs participants used in the branded pack condition, the lack of these elements on the plain packs may have increased participants’ negative attitudes towards the plain packs. The present study avoids this transfer of cigarettes and also any problems associated with participants using cigarette packs created by the researchers, as both the packs used are tobacco industry packaged cigarettes.

There are also some limitations to our research design. First, although the plain packs from Australia and the branded packs from the UK are matched for brand, there are some differences between the packs such as the size and format of the health warnings, the constituent information on the pack, the design of the cigarettes themselves, and the specific tobacco in the cigarettes. However, given that plain packaging legislation will most likely be introduced alongside larger health warnings similar to those on the Australian plain packs, using these plain packs increases the ecological validity of the study. Second, the smokers in this study will be more familiar with the UK cigarette packages and it is possible that any reduction in cigarette consumption observed in this study may be attributable to this reduction in familiarity rather than a perceived reduction in value of the cigarettes. As the current study will be conducted over a short time-frame, with participants only using the cigarettes provided to them for 24 hours, the present study cannot distinguish between these two possibilities. However, if a significant reduction in consumption were observed, this would justify a longer trial to determine whether this reduction is sustained over the longer-term; a result which would not be easily attributable to familiarity or design confounds. A longer-term trial design was not chosen for the present study as this would have resulted in difficulties in maintaining compliance with the use of the monitors, an important aspect of this study. By using the topography monitors to measure smoking behavior, our method is far more sensitive to small changes in smoking behavior, such as the volume of smoke inhaled, than self-report measures.

This study is novel in its approach to assessing the impact of plain packaging on actual smoking behavior. The research will help inform policymakers about the effectiveness of plain packaging as a tobacco control measure.

### Trial status

This article was first submitted on 18 November 2013 and was resubmitted on 27 May 2014. The first participant was enrolled in March 2013 and the last participant was enrolled on 11 December 2013.

## Abbreviations

ANOVA: Analysis of variance; FTND: Fagerström test for nicotine dependence; QSU: Questionnaire of smoking urges; SD: Standard deviation; UK: United Kingdom.

## Competing interests

The authors declare that they have no competing interests.

## Authors’ contributions

OMM, UL, LB and MRM were responsible for the conception and design of the trial, and the plan for the analysis of the data. LH was responsible for the design of the cigarette reward task. OMM participated in data collection, was responsible for the recruitment and treatment of participants, and wrote the first draft of the manuscript. ASA was responsible for participant randomization and assisted with statistical analyses. All authors discussed, read and revised the manuscript, and approved the final version of this manuscript.
